# Inter-MAR Association Contributes to Transcriptionally Active Looping Events in Human *β-globin* Gene Cluster

**DOI:** 10.1371/journal.pone.0004629

**Published:** 2009-02-27

**Authors:** Li Wang, Li-Jun Di, Xiang Lv, Wei Zheng, Zheng Xue, Zhi-Chen Guo, De-Pei Liu, Chi-Chuan Liang

**Affiliations:** National Laboratory of Medical Molecular Biology, Institute of Basic Medical Sciences, Chinese Academy of Medical Sciences (CAMS) and Peking Union Medical College (PUMC), Beijing, People's Republic of China; University of Munich and Center of Integrated Protein Science, Germany

## Abstract

Matrix attachment regions (MARs) are important in chromatin organization and gene regulation. Although it is known that there are a number of MAR elements in the *β-globin* gene cluster, it is unclear that how these MAR elements are involved in regulating *β-globin* genes expression. Here, we report the identification of a new MAR element at the LCR(locus control region) of human *β-globin* gene cluster and the detection of the inter-MAR association within the *β-globin* gene cluster. Also, we demonstrate that SATB1, a protein factor that has been implicated in the formation of network like higher order chromatin structures at some gene loci, takes part in *β-globin* specific inter-MAR association through binding the specific MARs. Knocking down of SATB1 obviously reduces the binding of SATB1 to the MARs and diminishes the frequency of the inter-MAR association. As a result, the ACH establishment and the *α*-like *globin* genes and *β*-like *globin* genes expressions are affected either. In summary, our results suggest that SATB1 is a regulatory factor of hemoglobin genes, especially the early differentiation genes at least through affecting the higher order chromatin structure.

## Introduction

Many studies have shown that a transcriptionally active structure can be formed when genes express actively. Several assays including 3C [1], ChIP-3C/Loop assay [2], [3], DamID [4] and other approaches have been applied to show that the chromatin looping events can bring distal regulatory elements and related gene promoters into close proximity and provide such a transcriptionally active structure. The looping events have been documented on several gene loci such as *HBB* [5]–[9], *Igf2-H19* [10]–[12], *Igk* [13], *Dlx5/Dlx6* [3], *HoxB1* [14], and *TH2* loci [15], [16].

During exploring the possible formation mechanisms of spatial organization of chromatin in the nucleus, the Matrix/scaffold attachment regions (MARs/SARs) have been suggested to be the important players for the complex packaging of eukaryotic chromosomes in nuclei. MARs are originally identified as genomic DNA fragments that remain tightly associated with high salt-extracted and DNase I–digested nuclei, and have been postulated to be localized at the base of chromatin loops. The MARs help to form the chromatin loops by attaching to the nuclear matrix. MARs identified by such criteria often contain base-unpairing regions (BURs) which become continuously unpaired when subjected to negative super helical strain [17], [18]. SATB1, which has been characterized as a MAR-binding protein, can bind to the BUR sequences and regulates higher order chromatin loop structures in T-cell[19]–[21]. Kumar et al also showed that SATB1 can recruit a regulatory complex that manages transcription by orchestrating dynamic chromatin-loop architecture in MHC class I locus [21].

The intensive studies in the *β-globin* locus have revealed that the local chromatin organization is one of the major players in the in vivo regulation of *globin* genes expression. There are 5 developmentally specific genes including embryonic (*ε*), fetal (*Gγ*, *Aγ*), and adult (*δ,β*) globins in the human *β-globin* gene cluster on chromosome 11. The locus control region (LCR) of *β-globin* gene cluster, containing 5 DNase I-hypersensitive sites (HS), locates in the far upstream region of the cluster and is able to enhance tissue-specific *β-globin* genes expression[22], [23]. In the *β-globin* locus, the actively expressing globin genes are in close proximity to LCR to form a specialized structure that was termed as ACH [7]. However, this higher order chromatin structure is not directly associated with gene transcription. Erythroid-specific transacting factors, such as EKLF, GATA-1, and FOG [8], [9] are indispensable factors to recruit the active *β*-like *globin* genes to the ACH. The details of how this ACH structure was established and maintained are still unclear. There are many candidate MARs in the *β-globin* cluster appear to be important in regulating the *β*-like *globin* genes expression[24]. MARs flanking the *ε*- or *γ-globin* genes or within the *β-globin* second intervening sequence (IVS2) have been proposed as regulatory elements for *globin* genes expression or hemoglobin switching [25]–[28]. But how these MAR elements regulate the gene expression is only addressed most recently. SATB1 was found to be present in the erythroid cells and the binding of SATB1 to some MARs can up-regulate the expression of *ε-globin* gene [29]. These studies indicate that SATB1 could mediate the function of MAR elements in directing the expression of *β-globin* genes.

In this report, we identified a new MAR element within the β-globin locus and observed the SATB1 mediated inter-MAR association by applying several approaches. We also demonstrated that the inter-MAR association plays an important role in regulating the expression of *β*-like *globin* genes possibly through influencing the establishment of the ACH(active chromatin harbor). In light of the global regulatory role of SATB1, our data suggest that SATB1 is possibly an important regulator of early erythroid differentiation by influencing the chromatin organization.

## Results

### The identification of a new MAR element in the *β-globin* cluster

**Figure 1:**
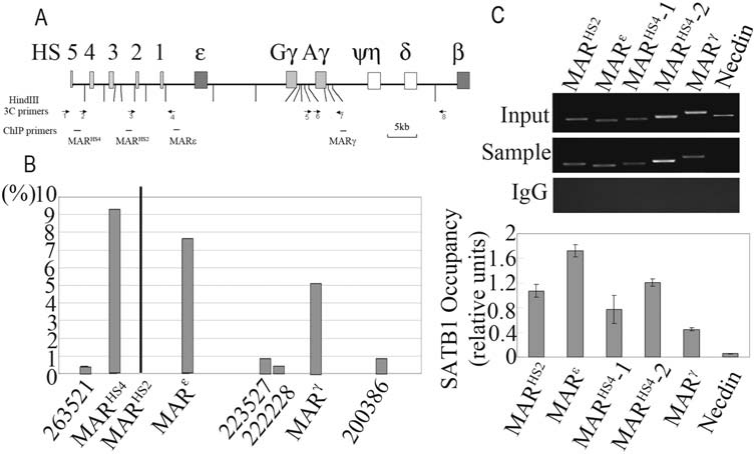
Identification of MAR^HS2^ associated chromatin fragments and SATB1 binding assay. A. Schematic representation of *β-globin* gene cluster is shown. The vertical lines represent the positions of HindIII digestion sites. The arrows represent the positions of the 3C primers and the numbers from 1 to 8 represent 263521, MAR^HS4^, MAR^HS2^, MAR^ε^, 223527, 222228, MAR^γ^ and 200386 respectively. The horizontal bars represent the positions of ChIP primers. B. The histogram shows the capturing frequencies (Y-value) of the chromatin fragments in QACT assay when MAR^HS2^ was used as the leader fragment. The MAR^HS2^ associated chromatin fragments identified by QACT including MAR^HS4^ and previously reported MAR^ε^ and MAR^γ^ were indicated along X-axis. The leader fragment (MAR^HS2^) of QACT assay was shown as the vertical line. Three representative low frequency fragments in β-globin cluster region were also shown and the numbers represent the sequence number in NT009237(NCBI). C. ChIP assay of SATB1 binding to MAR^HS4^(including 2 separate fragments MAR^HS4^-1 and MAR^HS4^-2), MAR^HS2^, MAR^ε^ and MAR^γ^. The positions of the ChIP primers are shown in Fig. 1A. The Y value is the enrichment of SATB1 after normalized to input. The histogram represents the average of triplicated experiments and the standard deviations were shown. Necdin was used as the negative control. An example of PCR-amplified products on 2% agrose gel is shown at the top.

We applied an improved method QACT (quantitative associated chromatin trap) to search the spatially associated chromatin fragments with a previously identified MAR element, which locate upstream of HS2 (MAR^HS2^) in K562 cells [29], [30]. The method is based on the recently reported ACT method [12] and could quantitatively identify previously unknown chromatin fragments associated with a given chromatin fragment [31]. The MAR^HS2^ is a well known MAR element that had been proved to regulate the *β*-like *globin* genes expression [29]. There were totally 3 restriction fragments identified by QACT assay that show obvious association with the MAR^HS2^ (Fig. 1A and Fig. 1B). The fragments from the 5′-flanking region of *ε-globin* gene promoter and 3′ -flanking region of *γ^A^ -globin* gene contain the supposed potential MARs (MAR^ε^ and MAR^γ^) that have already been reported [22], [25], [26], [30], [32], [33]. Another restriction fragment that showed high association frequency with MAR^HS2^ locates at the interval region between HS4 and HS5. This newly identified fragment showed high MAR potential when analyzed with MARwiz (Fig. S4A) [34]. The nuclear extraction/DNA retention assay that is used to prove the enrichment of this fragment in NM(nuclear matrix) associating chromatin also confirmed the high MAR possibility of this fragment (Fig. S4B)[28], [35], [36]. SATB1 specifically binds to double stranded BUR sequences by recognizing a specialized DNA context (an ATC sequence context), that frequently characterizes the DNA content of MARs [37]. To test the MAR potential of this newly identified fragment, the core sequence of this fragment with predicted SATB1 binding sites was detected for the SATB1 binding capacity by EMSA. Compared with the well-known SATB1 binding sequence [29], [37], the tested sequence showed similar binding capability (data not shown). In addition, the ChIP result also showed an obvious in vivo binding of SATB1 to the two core sequences of this region (Fig. 1C). Therefore, both the in vitro and in vivo experiments proved that the newly identified fragment has SATB1 binding capability, suggesting that a potential MAR is enclosed in the interval region between HS4 and HS5. Here, we name it as MAR^HS4^.

### MARs in *β-globin* gene cluster associate with each other

**Figure 2:**
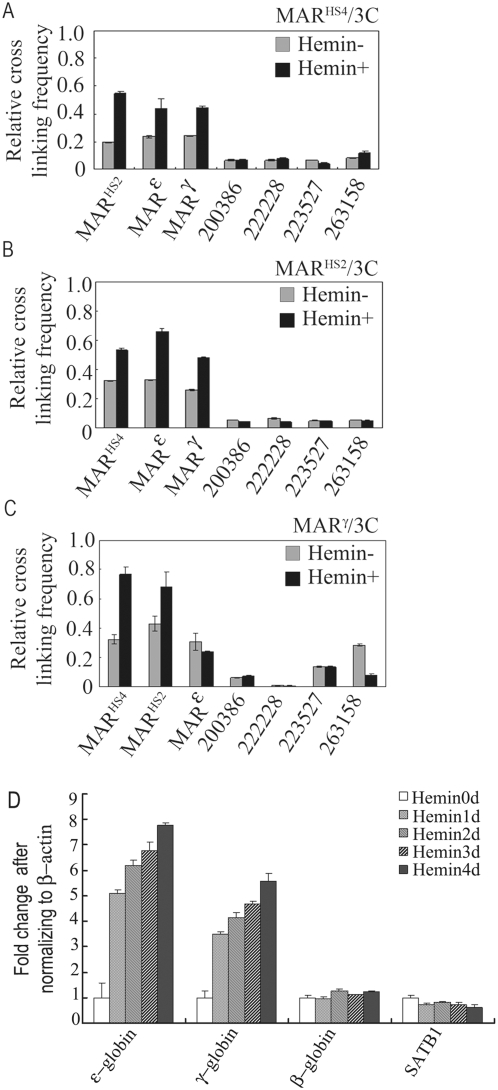
3C assay of MAR associations and the expressions assay of *β*-like *globin* genes in both hemin induced and uninduced K562 cells. The histograms show the association frequencies between the leader fragment and other tested fragments. The leader fragments are MAR^HS4^ (A), MAR^HS2^ (B) and MAR^γ^ (C) respectively. The tested fragments are shown along the X-axis and the leader is shown at top-right. Four fragments from other regions of *β-globin* gene cluster were also analyzed. The positions of the primers are shown in Fig. 1A. The Y values of the histograms represent the reading of PCR signal of two-tested fragments ligation product after normalization, which represent the ligation frequency of each pair of analyzed fragments (see materials and methods for details). The PCR-amplified re-ligation product from GAPDH locus was used to correct for the amount of DNA (the 3C templates DNA prepared from hemin uninduced K562 cells and hemin induced K562 cells) used in each PCR. Error bars represent the mean and standard errors from triplicate and averaged determinations. D. Quantified assay of the expressions of *β*-like *globin* genes during the hemin induction after normalizing to β-actin.

In the QACT assay, MAR^HS2^ was extensively associated with MAR^HS4^, MAR^ε^ and MAR^γ^. To further validate the associations among these MAR elements, 3C assay was performed using MAR^HS4^, MAR^HS2^, and MAR^γ^ located restriction fragments as the fixed fragment respectively. The results showed that the four MAR elements in *β-globin* gene cluster can associate with each other at obviously high frequencies (Fig. 2A, B, C), suggesting that the four MAR elements are spatially close to each other. The association results in the looping out of the intermediate regions between different MARs. This observation is consistent with the hypothesis that genomic DNA is attached to the NM through MAR elements and the intermediate regions between two MAR elements are organized as separate units. Also, the positions of these four MAR elements on NM are spatially proximal which indicates a “MAR core” structure in the *β-globin* gene locus in K562 cells. The functional significance of this “MAR core” could be observed when the terminal differentiation of K562 cells was induced by hemin. More than 90% cells could be induced when measured by, the Benzidine staining(Fig. S1) [38]–[41]. The comparative 3C assay in uninduced and induced K562 cells showed the obvious increasing of the ligation frequencies among the MARs after hemin induction (Fig. 2A, B, C), implying that the “MAR core” formation is also significantly increased accompanying the up-regulation of the *β*-like *globin* genes expression (Fig. 2D). Taken together, these data indicate that the association of the four MAR elements of *β-globin* gene cluster establishes a “MAR-core” as the base to organize the intermediate regions. In addition, the association of the MAR elements can be induced by hemin and the association accompanies the activation of *ε* and *γ-globin* genes in K562 cells.

### SATB1 can specifically bind to these potential MARs and mediate the association among these MARs in *β-globin* gene cluster

**Figure 3:**
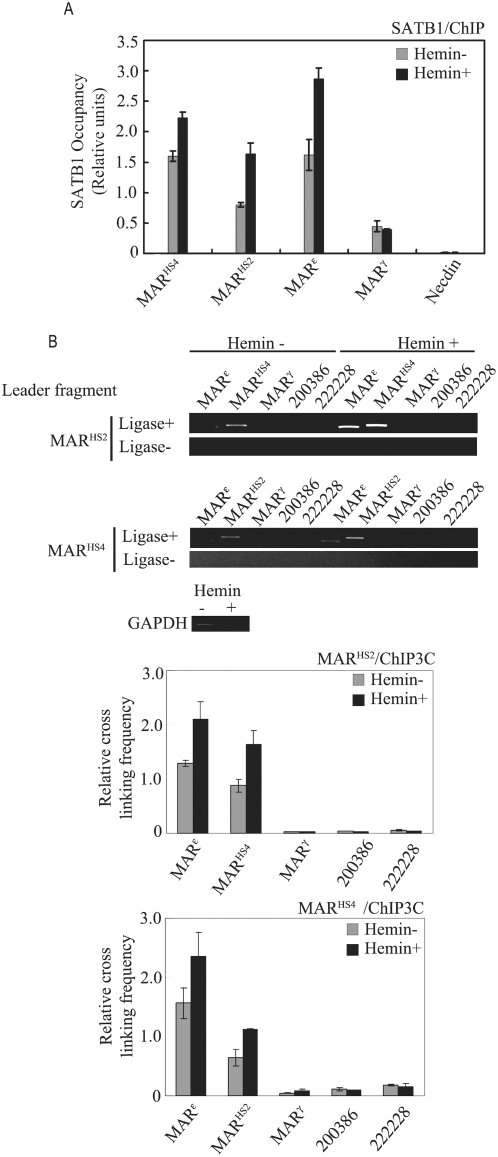
SATB1 mediated inter-MAR association. A. ChIP assay of the varying SATB1 binding at MAR elements during the hemin induced K562 cells differentiation. The four detected MAR elements are indicated along X-axis and Necdin is used as the negative control. The positions of the ChIP primers are shown in Fig. 1A. The Y-value represents the reading of PCR signal after being normalized to input. B. ChIP-3C analysis of inter-MAR association. The samples without ligase (ligase-) are used as negative control. The positions of the tested fragments were indicated at the top of each gel. The re-ligation frequencies of two adjacent GAPDH fragments were used to normalize the starting material of each assay. The histogram is to show three biological replicates of ChIP-3C assay and the error bars represent the standard deviations of these three replicates. The Y values represent the relative re-ligation frequencies indicated by gray and black bars between fragment MAR^HS2^ or MAR^HS4^ as the leader fragment and the other fragments of the locus.

The *β*-like *globin genes* expressions are always accompanied by the establishment of specific 3D chromatin structure [5], [7], [42]. The transcription factors are supposed to be responsible for this active process. SATB1 is one of the candidates that have been reported to regulate the *β-globin* genes expression by influencing the chromatin structure [29]. We compared the varying binding status of SATB1 at the MAR elements in *β-globin* cluster during the erythroid differentiation. As expected, we detected the strong binding of SATB1 to at least three of the four MARs including MAR^HS4^, MAR^HS2^ and MAR^ε^ in uninduced K562 cells (Fig. 3A). Hemin induction of the K562 cells obviously increased the binding of SATB1 to MAR^HS4^, MAR^HS2^ and MAR^ε^. Also worthy of noting is that the binding of SATB1 to MAR^γ^ was much weaker than other MARs regardless of the differentiation status (Fig. 3A). This is not consistent with the previous in vitro assay[26], but consistent with the in vivo binding assay reported recently [29]. The ChIP results also showed that there was no binding of SATB1 to the globin gene promoters including *ε*- and *γ-globin* genes or the core regulatory elements including HS2 and HS4 (Fig. S2), suggesting a binding specificity between SATB1 and MARs. This result indicates that the increased SATB1 binding is a possible reason of increased inter-MAR association when the polymerization property of SATB1 is taken into consideration [19]. To further explore the essential role of SATB1 in mediating the inter-MAR association, we applied the “ChIP-3C” assay in both uninduced and induced K562 cells. Compared with 3C assay, the ChIP-3C assay allows us to examine a specific chromatin interaction mediated by a particular protein [2], [3]. As the ChIP-3C result showed in both cells, the re-ligation products between any two of MAR^HS4^, MAR^HS2^ and MAR^ε^ can be observed consistently (Fig. 3B), indicating that SATB1 mediates the inter-MAR association at least among MAR^HS4^, MAR^HS2^ and MAR^ε^. Sequencing of the PCR products further confirmed the re-ligation products. We haven't seen any signal of re-ligation products between MAR^HS4^/MAR^HS2^ and MAR^γ^ or other nonspecific chromatin fragments from outside of MAR regions. However, we did observe the association between MAR^γ^ and MAR^HS4^/MAR^HS2^ as shown in 3C assay (Fig. 2), suggesting that the association is possibly mediated by other trans-acting factor(s). In fact, the binding of SATB1 to MAR^γ^ was marginal compared with others as shown in the ChIP assay (Fig. 1B). Additionally, Wen et al also reported that the increasing of *γ-globin* gene expression was accompanied by the decreasing of SATB1 expression after hemin induction in the late passage K562 cells [29], supporting that SATB1 is not a critical regulator of *γ-globin* gene expression. Additionally, the ChIP-3C result also supports that hemin induction of K562 cells significantly increases the association frequency of these 3 MARs including MAR^HS4^, MAR^HS2^ and MAR^ε^ (Fig. 3B). This result is consistent with the increasing binding of SATB1 to these 3 MARs and increasing association frequency among these 3 MARs as shown in Fig. 2.

### SATB1 is important for higher order chromatin organization and *β-globin* genes expression in K562 cells

**Figure 4:**
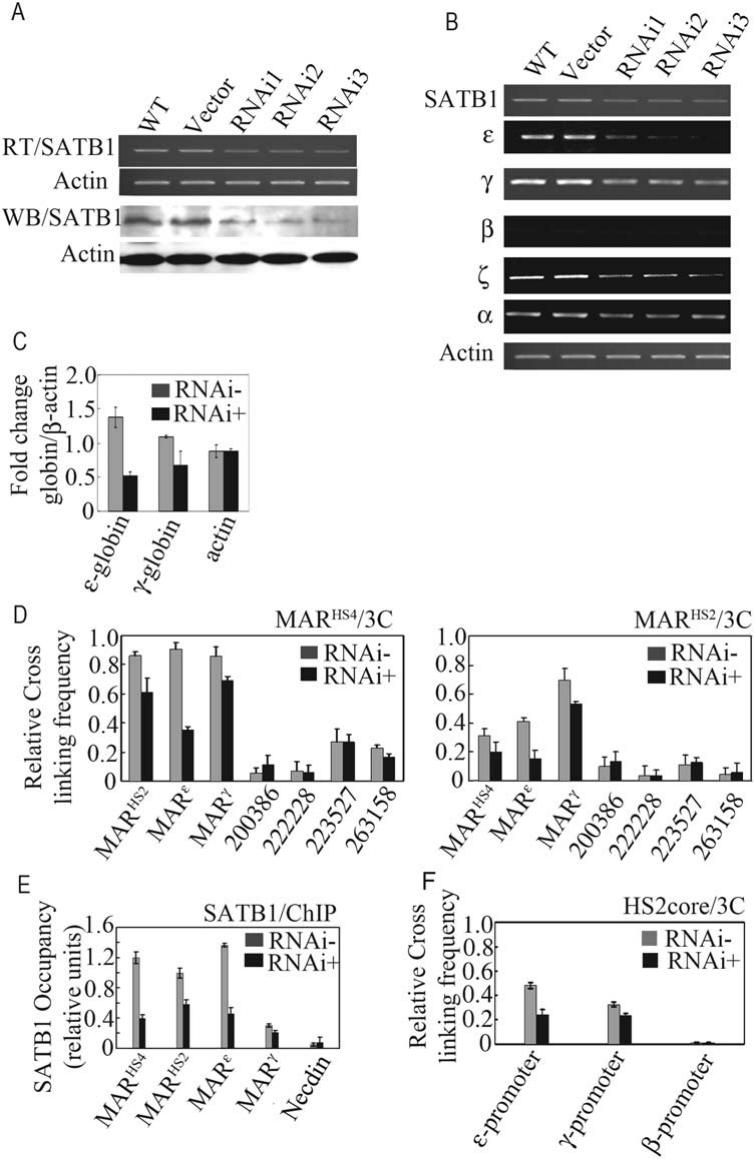
Knocking down of SATB1 has profound effect on the chromatin structure at *β-globin* gene locus. A. The detection of the SATB1 level by RT-PCR and western-blotting after SATB1 being knocked down through RNA interference(RNAi). RNAi1, RNAi2 and RNAi3 represent 3 independent SATB1-knockdown K562 cell clones. The vector with non-specific RNAi cassette was used as negative control and the wild type (WT) K562 cell was used as positive control. B. The expressions of *ε-globin*, *γ-globin*, *β-globin*, *α-globin* and *ζ-globin* genes were determined by semi quantitative RT-PCR and normalized to *β–actin* in both wild type K562 cells and SATB1-knock-down cells RNAi3. C. The histogram is to show three biological replicates of the expression assay of *ε-globin* and *γ-globin* genes by real-time PCR. D. 3C assay of inter-MAR association in wild type K562 cells and SATB1-knockdown cells RNAi3 with MAR^HS4^ and MAR^HS2^ as leader fragment respectively. E. ChIP assay of SATB1 binding to MARs in both wild type K562 cells and SATB1-knockdown RNAi3 cells. F. 3C assay of the spatial proximity between HS2 and *ε-globin*, *γ-globin* or *β-globin* genes promoters. HS2core represents the core fragment of HS2 after HindIII digestion which was used as the leader fragment. Error bars in C, D, E, and F represent the mean and standard error from triplicate and averaged determinations.

**Figure 5:**
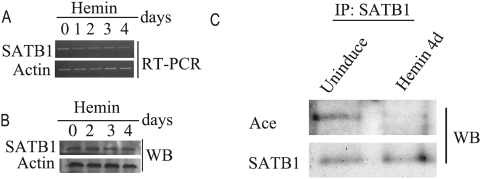
Expression and modification assay of SATB1 after hemin induction. The detection of SATB1 expression during the hemin induction by RT-PCR(A) or Western-blotting(B). C. Acetylation analysis of SATB1 after hemin induction for 4 days. The Anti-SATB1 antibody immunoprecipitated proteins were detected by either Anti-SATB1 antibody or Acetylation specific antibody. The wild type K562 cells(uninduced) were used as the control.

We also applied the SATB1 specific RNAi vector that expresses a short hairpin RNA against SATB1 mRNA *in vivo* to detect the role of SATB1 in mediating establishment of the inter-MAR association. We transfected K562 cells with the SATB1-RNAi vector or the control vector, and obtained the stably transfected cells [43]. It could be observed that the expression of SATB1 was markedly repressed in both mRNA and protein level (Fig. 4A). As a result, the substantially reduced expression of *ε-globin* gene could also be observed in the K562-SATB1-RNAi cells but not in the K562-SATB1-control cells (Fig. 4B, C). The expression of *β-globin* gene was unaltered compared with K562-SATB1-control cells. The expression of *γ-globin* gene was also moderately reduced (Fig. 4B, C). These results suggest that knocking down of SATB1 in K562 cells may influence the spatial chromatin structure of *β-globin* gene cluster. It is also noticeable that the *ζ-globin* gene, one of the *α*-like *globin* genes predominantly expresses in fetal stage was also obviously repressed in the K562-SATB1-RNAi cells(Fig. 4B), suggesting a general role of SATB1 in erythroid differentiation. Interestingly, there were no significant expression changes of some important erythroid transcriptional factors in the K562-SATB1-RNAi cells(Fig. S3). As expected, the bindings of SATB1 at MAR^HS4^, MAR^HS2^ and MAR^ε^ were reduced substantially in SATB1 knocking down cells, whereas the SATB1 binding at MAR^γ^ was still marginal (Fig. 4E). Accordingly, the 3C assay result showed that the association between MAR^HS4^/MAR^HS2^ and MAR^ε^ also obviously decreased (Fig. 4D). Additionally, the active transcriptional structure formed between HS2 core sequence and *ε-globin* gene promoter was affected in K562-SATB1-RNAi cells and the association between HS2core and *γ-globin* gene promoter also moderately decreased (Fig. 4F). These results suggest that SATB1 possibly contributes to the *γ-globin* expression. However, Wen et al has reported that knocking down of SATB1 resulted in the increasing of *γ-globin* gene expression in late passage of K562 cells, suggesting that SATB1 is not an important regulatory factor of γ-globin gene expression[29]. This discrepancy is possibly caused by the using of different passages of K562 cells. There are both early passage of K562 cells and late passage of K562 cells. In the early passage of K562 cells, the expressions of both *ε* and *γ-globin* genes increase in response to hemin induction as we observed in our experiments and other previous reports[38]–[40]. The amount of SATB1 in the early passage of K562 cells we used showed no obvious change after induction (Fig. 5A). While in the late passage of K562 cells used by Wen, et al., *ε-globin* gene decreased and *γ-globin* gene increased after hemin induction. Also, SATB1 decreased in response to the hemin induction. In our results, SATB1 knocking down probably generated more direct and significant repressing influence on *ε-globin* gene expression than on *γ-globin* gene expression in the early passage of K562 cells (Fig. 4B, C). However, the decreased expression of SATB1 in the late passage K562 cells could up-regulate the expression of *γ-globin* gene but down-regulate the expression of *ε-globin* gene, indicating the different regulatory patterns between these two subtypes of K562 cells.

### The acetylation modification of SATB1 changes in differentiated erythroid cells

As we observed, hemin induction of K562 cells could enhance the inter-MAR association and the expression of *globin* genes. Unexpectedly, *SATB1* gene expression showed no increase in both mRNA level and protein level during the hemin induction of K562 cells (Fig. 5 A, B). Because a recent report found that SATB1 could be acetylated in Jurkat cells in vivo and this acetylation significantly impaired the DNA binding ability of SATB1 [44], we performed the immunoprecipitation assay with SATB1 specific antibody to identify if SATB1 also could be acetylated in K562 cells. As shown in Fig. 5C, SATB1 that was precipitated by anti-SATB1 antibody could be recognized by acetylated-lysine specific antibody. Importantly, the acetylated SATB1 obviously decreased concomitant with the hemin induction of K562 cells. This result indicates that the equilibrium between the deacetylated and acetylated SATB1 could be affected by the hemin induction through promoting the deacetylation of SATB1 and increasing its DNA binding ability.

### SATB1 is possibly important for reinitiating expression of *β*-like *globin* genes during cell cycles

**Figure 6:**
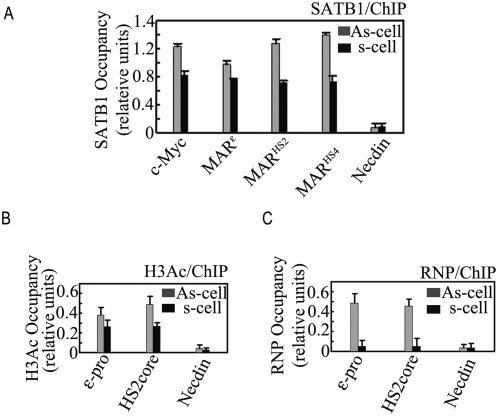
The binding of SATB1 at MARs of *β-globin* gene cluster in metaphase. The occupancy of SATB1(A), acetylated-H3 (H3Ac) (B) and RNP(C) at *β*-like *globin* gene clusters in asynchronous (As) and synchronous (s) K562 cells. The occupancies of SATB1 at target sites of *β-globin* clusters and *c-myc* gene in (As) and (s) cell populations were compared after normalizing the DNA amount differences in (As) and (s) populations through DNA input (In-As and In-s). Necdin was chosen as negative control of ChIP assay. The ε-pro indicates that the ChIP primer position is at the *ε-globin* promoter region. HS2core indicates that the ChIP primer position is at the core region of HS2. Error bars represent the standard deviations of at least 3 replicates.

From the above results, we concluded that knocking down of SATB1 mainly influences the inter-MAR association and the structural basis for *ε-globin* gene expression. Some studies suggested that the basic transcriptional factors like TFIID and TFIIB keep binding at active gene promoters in metaphase [45], [46]. This binding was supposed to preserve the transcription status of active genes. We wondered if SATB1 keeps binding at the MAR elements of the *β-globin* gene locus in metaphase. To answer this question, more than 90% K562 cells were synchronized into G2/M phases (Fig. S5) and ChIP was performed to observe the binding status of SATB1 at MAR^HS4^, MAR^HS2^ and MAR^ε^. As shown in Fig. 6A, the bindings of SATB1 at these three MAR sites were only decreased as mildly as what we observed on the histone 3 acetylation(H3Ac) (Fig. 6B) when the cells were synchronized into mitosis phase[47], whereas the RNPII almost lost the binding at both HS2 core sequence and *ε-globin* gene promoter(Fig. 6C), which is consistent with the abolishment of gene transcription during mitosis[48]. Taken together, these results demonstrate that SATB1 keeps binding at the MAR elements of *β- globin* gene cluster, implying that SATB1 may be involved in the reactivation of *β*-like *globin* genes during cell cycle.

## Discussion

The aim of our study was to show how MAR elements are involved in the gene regulation and how the MAR binding proteins influence the gene regulation through MAR elements using *β-globin* gene cluster in K562 cells as a model. By observing the positions of the MAR elements and the interactions among them, we showed the existence of a inter-MAR association structure mediated by SATB1 and the contribution of this structure to *β-globin* genes expression. We also observed the maintaining of the binding of SATB1 to these MARs, that is possibly important for inter-MAR association and the reactivation of genes transcription in next cell cycle. Therefore, our data, combined with other recent findings, imply a possibly new regulatory role of MAR elements when the gene regulation is depicted through the spatial chromatin organization level.

It is well known that the MARs/SARs are important players in complex packaging of eukaryotic chromosomes in nuclei by creating the chromatin loops attached to the nuclear matrix. For a long time, the nuclear matrix was proposed as a static structure that provides a platform or directly participates in diverse matrix-supported processes. Until recently, SATB1 mediated long range chromatin association was reported and this finding provided the first evidence that MAR elements are important regulatory elements helping to create the local looping structure [20], [21]. Within the *β-globin* gene cluster, several supposed MAR elements have been identified with different approaches [26], [29], [30], [49]. Most of the studies showed these MARs are able to attach to the nuclear matrix. In this study, we have identified the *in situ* association among these elements. Unexpectedly, a new MAR element, locating in the interval region between HS5 and HS4, has also been identified. We also proved that the MAR elements contribute significantly to the establishment of the local looping structure within *β-globin* gene cluster. However, there was also a report describing that the expression of *globin* genes were accompanied by the moving out of the whole locus from the chromosomal domain [50]. One of the suspected driving force was mediated by the attachment of MARs to nuclear matrix [28] or other subnuclear compartments. Therefore, the dual roles of MARs are also anticipated during the *β-globin* genes activation.

During the *β-globin* gene cluster activation, several transcriptional factors mediate the associations between the gene promoters and the enhancers [8], [9] to form active chromatin transcriptionally structure [5], [7] and our 3C assay has also confirmed that (Fig. S6). Our results have indicated that the *β-globin* gene cluster specific inter-MAR association is correlated with the formation of ACH. According to our 3C and ChIP-3C results, the association directly generates at least three intact loops, with HS3 and HS4 at one loop, HS1 and HS2 at another loop and *ε*- and *γ- globin* genes at the third loop. The three loops, therefore, provide the spatial convenience for the conversation among these HSs and the gene promoters. We presume that the formation of these loops is the structural basis for the establishment of ACH. Though there is no direct evidence for the relationship between the inter-MAR association structure and the previously described ACH structure, our data imply the co-existence of these structures mediated by different transcriptional factors. Also, our 3C assay showed that the association frequencys between MAR^HS4^/MAR^HS2^ and MAR^γ^ were higher than the frequency between MAR^HS4^/MAR^HS2^ and *γ-globin* promoter (Fig. S7). Importantly, our study showed that the inter-MAR association can increase substantially after hemin induction of K562 cells, that is consistent with the changed active looping events. Here, the results give a hint that the inter-MAR association structure may act as a pre-existing structure facilitating this activation process. Therefore, this structure should be relative stable and its establishment is not presumed to be mediated by the more dynamic transcriptional factor(s). However, it is only the beginning to realize the possible importance of inter-MAR association structure and much more efforts are required to further establish the role of the inter-MAR association at other gene loci.

SATB1 has the specific MAR binding property and can self-polymerize to establish a birdcage-like structure that could encapsulate the silenced genes [19]. SATB1 was also reported to mediate the long range chromatin associations in the TH2 cytokine locus, suggesting that the polymerization of SATB1 generates stable structure besides the binding to chromatin fragments as a transcriptional factor [20]. We also proved that SATB1 is important in the establishment of active loop formation between ε-promoter and regulatory elements. The associations among MAR^ε^, MAR^HS2^ and the newly identified MAR^HS4^, can be consistently observed and be proved to be mediated by SATB1. Because the signal between MAR^γ^ and MAR^HS4^/MAR^HS2^ can not be detected by ChIP-3C, we speculate that SATB1 is not a necessary factor that mediates the recruitment of *γ-globin* gene to the ACH. Because MAR^γ^ can be observed to be associated with the other three MARs, we also presume that it is mediated by an unknown factor. Looping events have been observed to be mediated by distinct transcriptional factors[51], which may confer the loops different regulatory functions and generate distinct outcomes. Chromatin loops formed by CTCF are known as independent regulatory units be protected from the surrounding cis-elements [52]. While SATB1, as suggested by our present data, may involved in the establishment of separate chromatin loops that can easily communicate with each other. Additional investigation will be necessary to propose a general partition in the function of different loops events.

There are evidences supporting that most of the basal transcription factors, RNA polymerases and enhancer binding factors are absent from the condensed, mitotic chromosomes [53], whereas the epigenetic markers of active and inactive genes can be maintained throughout mitosis, as well as the basal transcriptional factors like TFIID and TFIIB, which will not be excluded from active gene promoters during mitosis [45]–[47], [54], [55]. This maintaining is thought to be important for preserving the active status of active genes. However, the reactivation of gene expression also depends on the re-establishment of the active transcribing structure. Although there is no direct evidence to prove that the ACH structure breaks down during cell division, the enhancer-promoter interaction was observed to be lost in metaphase[56]. Also, the transcriptional machinery and several gene specific transcriptional factors required for the ACH formation are absent during metaphase[9], [45], [47]. Therefore, we presume that the re-establishment of ACH is possibly needed for the *β*-like *globin* genes expression and SATB1 binding at the MAR elements probably contributes to this active process.

**Figure 7:**
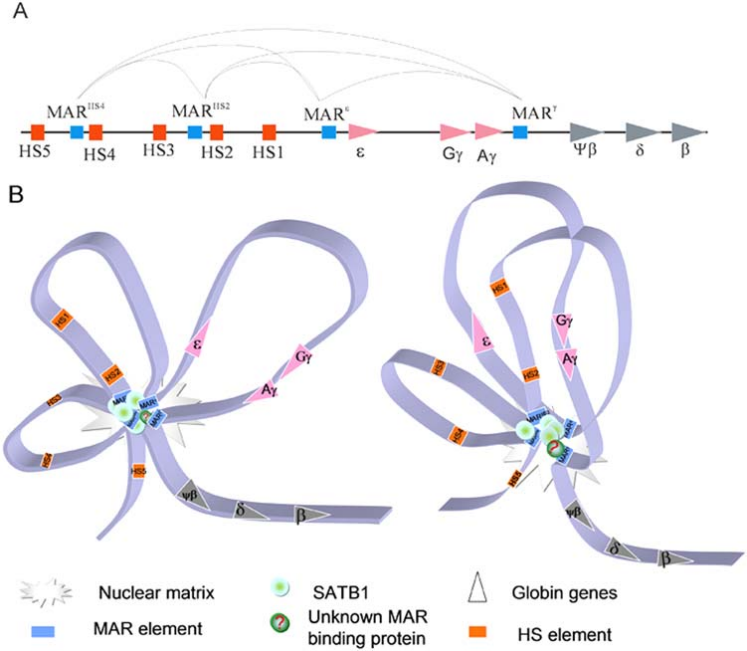
The MAR-core-loop model. A. The diagram illustrates the genomic arrangement of *β-globin* gene cluster. The orange squares represent HS1 to HS5 and the blue squares represent the MAR elements. The triangles represent the *β*-like *globin* genes. The curves at the top indicate the interactions among the four MAR elements. B. The supposed spatial organization of *β-globin* gene cluster during terminal differentiation of K562 cells, a “MAR-core-loop” model. The green spheres represent SATB1 which mediates the association of MAR elements. It is noticeable that the MAR^γ^ is possibly recruited to the “MAR core” by unknown MAR binding protein(s). Spatial proximity of the loops provides the chance for establishment of the ACH. The left panel shows the loops extending from the “MAR-core” and the right panel shows the loop interactions mediated by “MAR-core”.

As a conclusion, here we propose a “MAR-core-loop” model for better interpreting the function of SATB1 mediated inter-MAR association in K562 cells (Fig. 7). The positions of these MAR elements divide the whole *β-globin* gene cluster into at least 5 parts in K562 cells (Fig. 7A). Establishment of “MAR core” by MAR^HS4^, MAR^HS2^, MAR^ε^ and MAR^γ^ interactions generates at least 3 separated loops with which HSs, *ε-globin* and *γ-globin* gene promoters harbored. SATB1 is supposed to be the dominant mediator of the inter-MAR association. A predicted unknown factor is also involved in mediating the MAR^γ^ association to other members of this “MAR core”. As shown in Fig. 7B, the “MAR-core-loop” structure is a stable higher order chromatin structure that provides the structural convenience for the crosstalk among HSs or between HSs and gene promoters to form an active transcriptional structure termed as ACH. Importantly, the binding of SATB1 at MAR^HS4^, MAR^HS2^ and MAR^ε^ during the mitotic cell division helps to reactivate *β-globin* genes and high order chromatin structure during cell cycles.

## Materials and Methods

### Quantitative ACT(QACT)

QACT is designed based on the recently reported ACT method that could quantitatively identify the long distance associated chromatin fragments to a given chromatin region[31]. Briefly, the prepared 3C templates DNA were completely digested by the secondary endonuclease MspI and then purified. The digested DNA was used for self-ligation in 500 ul to promote the intra-molecule ligation. The primers used for nested inverse PCR were as follows: FMAR^HS2^F:CACTGAAAATAGTGTTTAGCAT; FMAR^HS2^ R: GTATCTTATTCCCCACAAGAGT; SMAR^HS2^F: TCCAGCATCCTCATCTCTGA; SMAR^HS2^R: ACAGTTAATTATAATGTGCTCTGTC. The 5′ side of SMAR^HS2^ primers was modified with biotin. PCR reactions were performed as follows: one cycle at 94°C for 4 min; 25 cycles at 94°C for 30 s, 56°C for 40 s and 72°C for 30 s; followed by one cycle at 72°C for 10 min. The first round PCR products were used as the templates of second round inverse PCR after 100 fold dilution. The second round PCR was performed as follows: one cycle at 94°C for 4 min; 30 cycles at 94°C for 30 s, 60°C for 40 s and 72°C for 30 s; followed by one cycle at 72°C for 10 min. The leader RCF was removed from the purified second round PCR products and the HindIII adaptor was ligated to the left ACPs. HindIII adaptor was obtained by annealing the following primers: HindIII adaptorF 5′ Biotin-ATACGACTCATGGATCCGACA; HindIII adaptorR 5′ Phosphate-AGCTTGTCGGATCCATGAGTCGTAT. BamHI and MmeI restriction recognition sites were included in the HindIII adaptor. Further MmeI digestion excised 19/20 bp tags from the ACPs and the tags could be captured by magnetic sphere through the HindIII adaptor and its biotin modification. To quantitatively analyze the possible associated chromatin fragments,a NN adaptor was ligated to the other side of the tags. NN adaptor was obtained by annealing of the following primers: NNadF: 5′ GCAAGGTGCTCTGCTGCAGNN; and NNadR: 5′ phosphate-CTGCAGCAGAGCACCTTGC. A PstI restriction site was included in the NN adaptor. Tags were amplified by additional PCR reaction with HindIIIadF and NNadF as primers. The two sides of adaptor were removed by BamHI and PstI.. Self-ligation of these tags produces concatemers including several to nearly twenty tags. The concatemers around 500 bp in length were cloned directly into the MCS(multi cloning site) of the vector pUC19. The inserted concatemers were sequenced. A frequency can be showed according to the sequencing results. The sequences of the tags could be mapped to the human genome with the BLASTn tool.

### Nuclear extraction/DNA retention

The nuclear extraction/DNA retention assay was performed as previously described[28], [35], [36]. In brief, The K562 cells were lysed on ice for 15 min using nuclei buffer to get nuclei.Nuclear extraction (Halo nuclei) were prepared by extracting nuclei with halo buffer and isolated by centrifugation through a glycerol step-gradient. The restriction endonucleases were added (∼100 U each, EcoRI/HindIII) and the DNA digested at 37°C for 4 h. The nuclear matrix(NM) DNAs were separated from the loop-associated(LA) DNA by centrifugation at 16,000×g for 20 min. Both NM and LA DNAs were isolated by reverse cross linking and purified by extraction with phenol and chloroform. Both samples were then subjected to 0.8% agarose gel electrophoresis and Southern hybridization was performed.

### 3C (chromosomal conformation capture)assay

The 3C assay was performed as previously described[1], [5] with a few modifications[57]. Firstly, we chose four representative sites at MAR^HS4^, MAR^HS2^, MAR^ε^, MAR^γ^ and some gene coding regions to perform the agarose gel electrophoresis after HindIII digestion(Fig. S8). RealtimePCR and semi-quantitative PCR with primers encompass several Hind III digestion sites to show that the digestion efficiencies at different sites are similar(Fig. S9). The β-globin BAC clones used to correct for the PCR amplification efficiency is screened from BAC library in our Lab(BAC 186D7) and the vector is pBeloBAC11. Primer sequences are available when required, GAPDH-1 (GCCCAATACGACCAAATCTAA) and GAPDH-2 (ATTGTTGCCATCAATGACCC) are the primers from two *HindIII* restriction fragments of the GAPDH gene used for correcting different template amount. All the test primer pairs were verified by amplifying the control sample and sequencing the PCR products. All the PCRs were triplicate and averaged. The correction method is the same as that given in the work of Dekker et al.[1] and Tolhuis et al[5]. The calculation gives a relative ligation frequency for each analyzed sample, since it corrects for the differences in PCR amplification efficiencies, amounts of templates, and sizes of PCR products.

### ChIP assay

ChIP analysis was carried out in hemin induced and uninduced K562 cells or SATB1 RNA interference(RNAi) cells. In brief, isolated, 1% formaldehyde-cross-linked cells, as described above, were lysed in lysis buffer (1% SDS, 10 mM EDTA, 50 mM Tris, pH 8.1) containing protease inhibitors and sonicated on ice until cross-linked chromatin DNA was sheared to an average length around 500 bp. The sonicated cell supernatant was diluted 10-fold in ChIP dilution buffer (0.01% SDS, 1.1% Triton X-100, 1.2 mM EDTA, 16.7 mM Tris–HCl, pH 8.1, 167 mM NaCl). The precleared chromatin using protein G-agarose (Upstate) was incubated with anti-SATB1 antibody (sc-5990, Santa Cruz), anti-acetylated-H3(06-599 Upstate), anti-acetylated-H4(06-866, Upstate) or with pre-immune goat serum as the control, at 4°C overnight. Immunoprecipitates were recovered by incubation with protein G-agarose (Upstate) at 4°C for 2 h, followed by low-speed centrifugation. The washed pellets were reverse cross-linked. The DNA was extracted with phenol-chloroform/isoamyl alcohol (25∶24∶1), precipitated with ethanol, and used for PCR analysis.

### ChIP-3C assay

The ChIP-3C assay was performed as previously described [2]. In brief, the crosslinked chromatin was sonicated, digested with specific restriction enzyme HindIII overnight, and immunoprecipitated with anti-SATB1 antibodies coupled to protein G beads. The beads were then precipitated, resuspended in ligation buffer, and overnight ligation was performed. The beads were washed using RIPA buffer [58]and protein-DNA complexes were eluted using ChIP elution buffer [59]. The crosslinking was reversed at 65°C for 15 h and ligated DNA was purified. The PCR primers for amplifying ligated DNA were as described in supplemental data.

### CoIP (Coimmunoprecipitation) assay

K562 cell lyses was obtained in the RIPA buffer (50 mM Tris-HCl(pH 7.5),100 mM NaCl,2 Mm EDTA,1% NP40,1 mM EGTA, protease inhibitor). Nuclear extract (100 µg) was first precleared with rabbit-IgG(Ssanta Cruz) and protein A/G beads(upstate). And the precleared extract was then incubated with SATB1 antibody or preimmune serum and proteinA/G beads in 1.5 ml IP buffer (0.5% NP-40, 10 mM Tris HCl, 150 mM NaCl, 2 mM EDTA, 10% glycerol, protease inhibitor) at 4°C for 4–6 h. After a brief centrifugation, the pellet was washed in IP buffer 4–5 times at 4°C for 10 min, and the protein-antibody complexes were analyzed by Western blot analysis with specific antibodies.

### Knock down SATB1 by RNA interference in K562 cells

SATB1 specific and non-specific siRNA vector were from X Han and Y Sun[43]. The SATB1 shRNA comprised of: 5′-GCTGAAAGAGACCGAATATTTCAAGAGAATATTCGGTCTCTTTCAGC-3′. The non-specific shRNA sequences were: 5′-ACG TGACACGTTCGGAGAATTCAAGAGATTCTCCGAACGTGTCACGT-3′.K562 cells were transfected with SATB1 RNAi plasmids or control plasmids using an Lipofectamine™ 2000 (Invitrogen). Several stable clones were selected using G418(500 µg/mL; Gibco, Grand Island, NY).The extent of siRNA mediated inhibition of SATB1 was evaluated by Western blot analysis with specific antibodies.

### Over-expression of SATB1 in K562 cells

An SATB1 expression vector was constructed by fusing *SATB1* cDNA into pEGFP-N2 (Clontech, PaloAlto, CA) to give pEGFP/SATB1; accuracy was confirmed by DNA sequencing. For stable cell lines, pEGFP/SATB1 or pEGFP was transfected by Lipofectamine™ 2000 (Invitrogen) into K562 cells. Several Clones were selected using G418 (500 µg/mL; Gibco, Grand Island, NY). The extent of overexpression of SATB1 was evaluated by RT-PCR and Western blot analysis with specific antibody.

### Quantitative Real-Time PCR Analysis

Differences in DNA enrichment for ChIP samples and 3C samples were determined by real-time PCR by using the 7500 Real Time PCR System (ABI). The threshold was set to cross a point at which PCR amplification was linear, and the number of cycles (Ct) required to reach the threshold was collected and analyzed with Microsoft Excel. Primers sequences are available upon request. The PCR product was measured by SYBR green fluorescence.

### Immunofluorescence

Immunofluorescence was carried out on synchronized K562 cells as described previously [47].

## Supporting Information

Figure S1 The Benzidine staining assay for uninduced and induced K562 cells by Hemin. A.The uninduced K562 cells; B. The induced K562 cells by hemin (4d)
(0.15 MB TIF)

Figure S2 The SATB1 binding analysis of MAR^HS2^, ε-globin promoter(ε-pro), γ-globin promoter(γ-pro), HS2Core and HS4Core elements by ChIP.
(0.21 MB TIF)

Figure S3 Expressions of GATA1, GATA2, CTCF, EKLF, NFE2 and MAFK were determined by normalized to β-actin in both wide-type K562 cells and SATB1-knockdown cells. A. Example of gel electrophoresis of PCR result. B. Average of triplicate experiments, error bars represent the standard deviations.
(0.29 MB TIF)

Figure S4 A. The analysis of MAR potential of MAR^HS4^ by MARwiz, a web-based analysis tool; B.Nuclear extraction/DNA retention assay of MAR^HS4^. The nuclear extraction was digested by the restriction enzyme(HindIII and EcoRI) and nuclear matrix associated fraction(NM) and loop associated fraction(LA) samples were separated by electrophoresis and hybrided by the MAR^HS4^ probe(2.6 kb). Genomic DNA digested by the same restriction enzyme(HindIII and EcoRI) was the positive control.
(0.19 MB TIF)

Figure S5 The synchronization of K562 cells after nocodazole treating. A. Asynchronous K562 cells population; B. synchronous mitotic K562 cells population.
(0.27 MB TIF)

Figure S6 Relative crosslinking frequencies between HS2core fragment as a leader and gene promoters including ε-pro, γ-pro and β-pro of the locus. The histogram shows the association frequencies between the leader fragment and other tested fragments. The tested fragments are shown along the X-axis and the leader is shown at top-right. The Y values of the histogram are the reading of PCR signal of two-tested fragments ligation product after normalization,, which represent the ligation frequency of each pair of analyzed fragments (see materials and methods for details). The PCR-amplified re-ligation product from GAPDH locus was used to correct for the amount of DNA (the 3C templates DNA from Hemin uninduced K562 cells and Hemin induced K562 cells) used in each PCR. Error bars represent the standard errors. “Hemin−” represents the uninduced K562 cells and “Hemin+” represents the induced K562 cells by Hemin.
(0.03 MB TIF)

Figure S7 Relative crosslinking frequencies between MAR^HS4^/MAR^HS2^ and MAR^γ^ were higher than the frequency between MAR^HS4^/MAR^HS2^ and γ-globin. A. The 3C assay using MAR^HS4^ and HS4 core fragments as the leader fragment. B.The 3C assay using MAR^HS2^ and HS2 core fragments as the leader fragment. The histogram shows the association frequencies between the leader fragment and other tested fragments. The Y values of the histogram are the reading of PCR signal of two-tested fragments ligation product after normalization,, which represent the ligation frequency of each pair of analyzed fragments. Error bars represent the standard errors.
(0.15 MB TIF)

Figure S8 The detection of enzyme digestion efficiency for 3C procedure template lane1 and 5. λ-Hind III marker; lane2 and 3. Hind III digested crosslinked genomic DNA (3C template); lane4.Hind III digested uncrosslinked genomic DNA.
(0.14 MB TIF)

Figure S9 Enzyme digestion efficiency detection. The histogram represents the analysis results from real-time PCR. Y-axis values represent relative enzyme digestion efficiency of 3C template, X-axis values represent primers that are designed to span one HindIII digestion site that is close to MAR^HS4^(primer1), MAR^HS2^(primer2), MAR^ε^(primer3), MAR^γ^(primer4), 263521(primer5), 223527(primer6), 222228(primer7), 200386(primer8) respectively.
(0.11 MB TIF)
