# Supplementary figures and images for: Inter-MAR Association Contributes to Transcriptionally Active Looping Events in Human β-globin Gene Cluster
Source: PLoS One. 2009 Feb 27;4(2):e4629. doi: 10.1371/journal.pone.0004629 (PMC2645683; doi:10.1371/journal.pone.0004629)

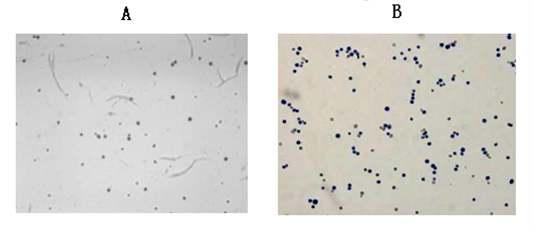

Supplement: Figure S1 — The Benzidine staining assay for uninduced and induced K562 cells by Hemin. A.The uninduced K562 cells; B. The induced K562 cells by hemin (4d) (0.15 MB TIF) [file pone.0004629.s001.tif]

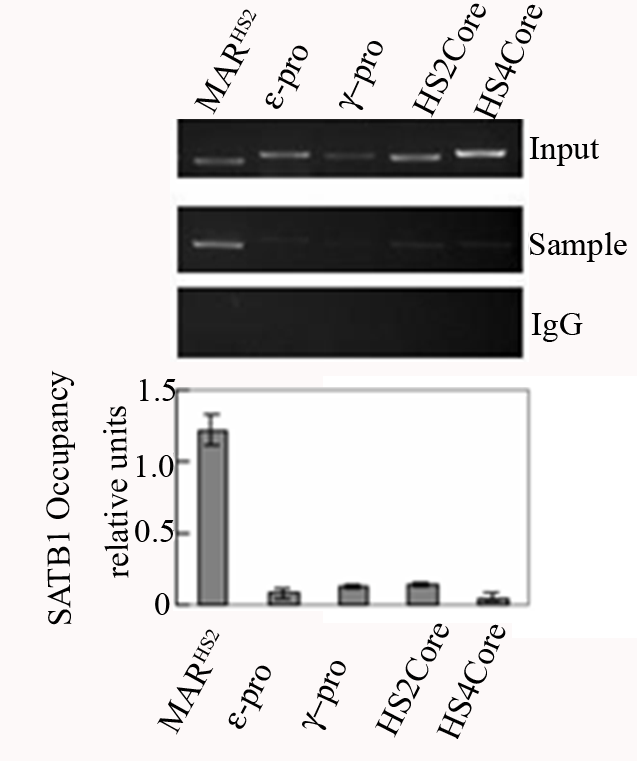

Supplement: Figure S2 — The SATB1 binding analysis of MARHS2, ε-globin promoter(ε-pro), γ-globin promoter(γ-pro), HS2Core and HS4Core elements by ChIP. (0.21 MB TIF) [file pone.0004629.s002.tif]

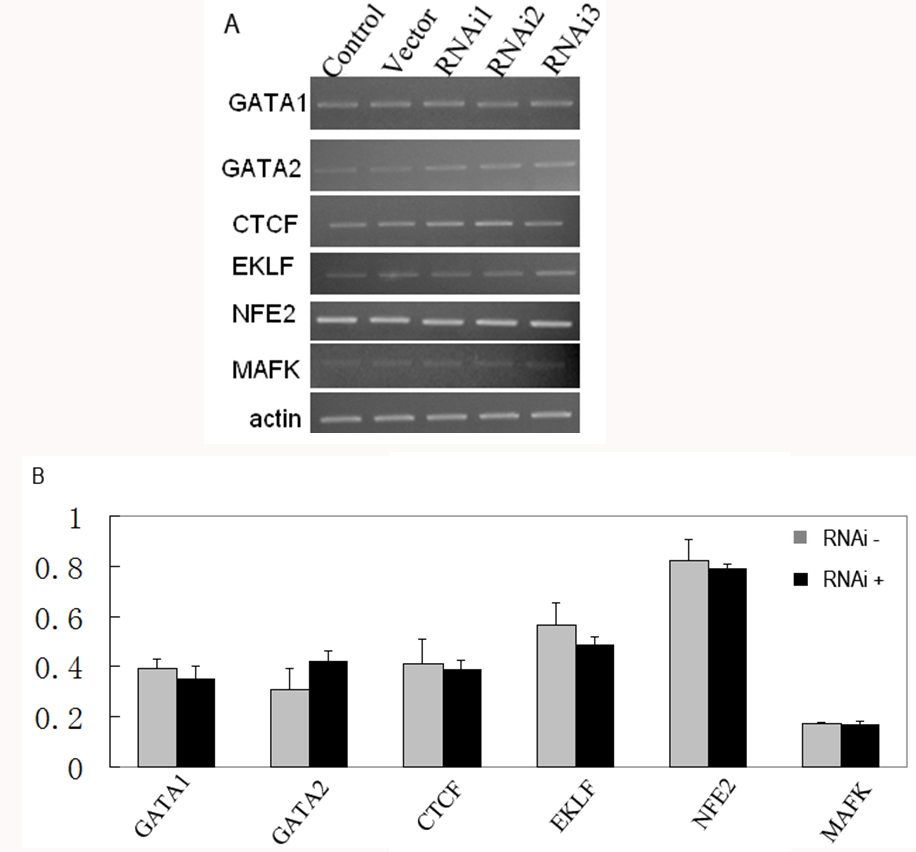

Supplement: Figure S3 — Expressions of GATA1, GATA2, CTCF, EKLF, NFE2 and MAFK were determined by normalized to β-actin in both wide-type K562 cells and SATB1-knockdown cells. A. Example of gel electrophoresis of PCR result. B. Average of triplicate experiments, error bars represent the standard deviations. (0.29 MB TIF) [file pone.0004629.s003.tif]

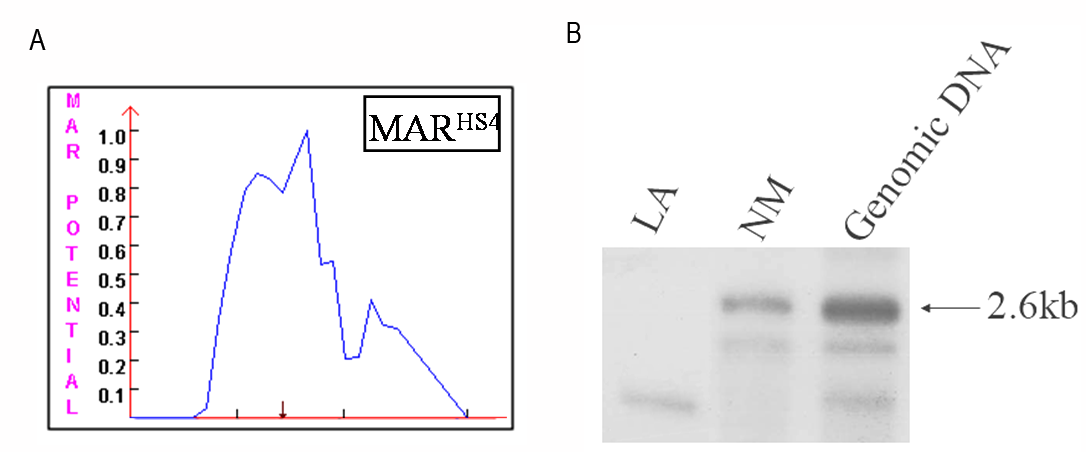

Supplement: Figure S4 — A. The analysis of MAR potential of MARHS4 by MARwiz, a web-based analysis tool; B.Nuclear extraction/DNA retention assay of MARHS4. The nuclear extraction was digested by the restriction enzyme(HindIII and EcoRI) and nuclear matrix associated fraction(NM) and loop associated fraction(LA) samples were separated by electrophoresis and hybrided by the MARHS4 probe(2.6 kb). Genomic DNA digested by the same restriction enzyme(HindIII and EcoRI) was the positive control. (0.19 MB TIF) [file pone.0004629.s004.tif]

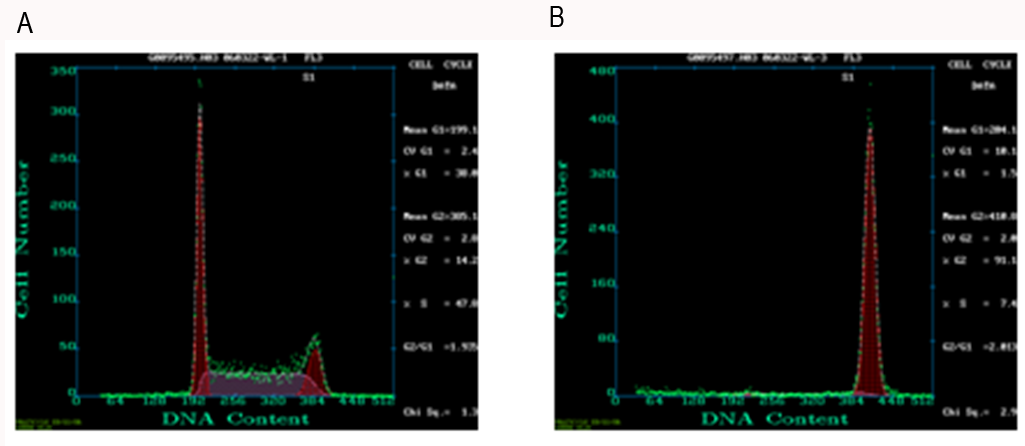

Supplement: Figure S5 — The synchronization of K562 cells after nocodazole treating. A. Asynchronous K562 cells population; B. synchronous mitotic K562 cells population. (0.27 MB TIF) [file pone.0004629.s005.tif]

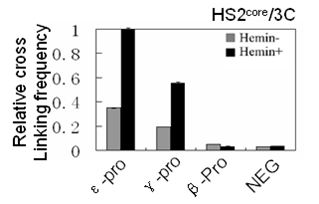

Supplement: Figure S6 — Relative crosslinking frequencies between HS2core fragment as a leader and gene promoters including ε-pro, γ-pro and β-pro of the locus. The histogram shows the association frequencies between the leader fragment and other tested fragments. The tested fragments are shown along the X-axis and the leader is shown at top-right. The Y values of the histogram are the reading of PCR signal of two-tested fragments ligation product after normalization,, which represent the ligation frequency of each pair of analyzed fragments (see materials and methods for details). The PCR-amplified re-ligation product from GAPDH locus was used to correct for the amount of DNA (the 3C templates DNA from Hemin uninduced K562 cells and Hemin induced K562 cells) used in each PCR. Error bars represent the standard errors. “Hemin−” represents the uninduced K562 cells and “Hemin+” represents the induced K562 cells by Hemin. (0.03 MB TIF) [file pone.0004629.s006.tif]

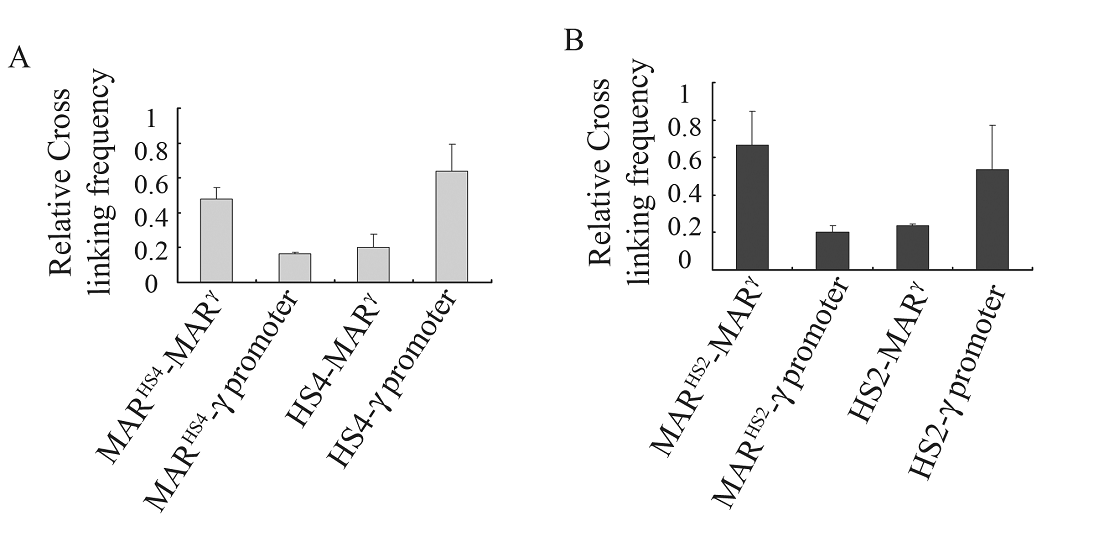

Supplement: Figure S7 — Relative crosslinking frequencies between MARHS4/MARHS2 and MARγ were higher than the frequency between MARHS4/MARHS2 and γ-globin. A. The 3C assay using MARHS4 and HS4 core fragments as the leader fragment. B.The 3C assay using MARHS2 and HS2 core fragments as the leader fragment. The histogram shows the association frequencies between the leader fragment and other tested fragments. The Y values of the histogram are the reading of PCR signal of two-tested fragments ligation product after normalization,, which represent the ligation frequency of each pair of analyzed fragments. Error bars represent the standard errors. (0.15 MB TIF) [file pone.0004629.s007.tif]

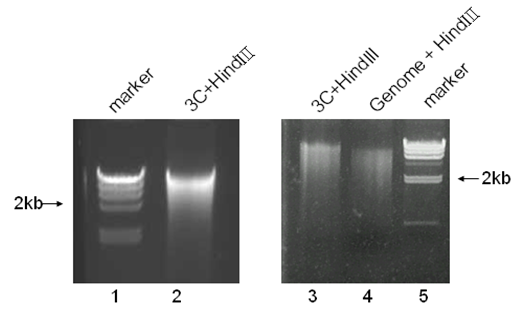

Supplement: Figure S8 — The detection of enzyme digestion efficiency for 3C procedure template lane1 and 5. λ-Hind III marker; lane2 and 3. Hind III digested crosslinked genomic DNA (3C template); lane4.Hind III digested uncrosslinked genomic DNA. (0.14 MB TIF) [file pone.0004629.s008.tif]

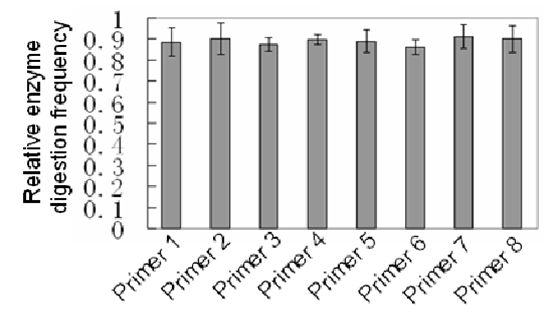

Supplement: Figure S9 — Enzyme digestion efficiency detection. The histogram represents the analysis results from real-time PCR. Y-axis values represent relative enzyme digestion efficiency of 3C template, X-axis values represent primers that are designed to span one HindIII digestion site that is close to MARHS4(primer1), MARHS2(primer2), MARε(primer3), MARγ(primer4), 263521(primer5), 223527(primer6), 222228(primer7), 200386(primer8) respectively. (0.11 MB TIF) [file pone.0004629.s009.tif]
